# Resurgence of Chikungunya virus: rising global threat and challenges in its mitigation

**DOI:** 10.3389/fphar.2026.1757035

**Published:** 2026-04-22

**Authors:** Priyadarshi Soumyaranjan Sahu, Ritesh Pattnaik, Mohammed Alissa, Ghadah S. Abusalim, Alaa S. Alhegaili, Ghfren S. Aloraini, Abdulkarim S. Binshaya, Ghada M. Alnafesah, Subrat Kumar

**Affiliations:** 1 Faculty of Pre-clinical Medicine, Medical University of the Americas, Nevis, West Indies; 2 School of Biotechnology, KIIT Deemed-to-be-University, Bhubaneswar, Odisha, India; 3 Department of Medical Laboratory, College of Applied Medical Sciences, Prince Sattam bin Abdulaziz University, Al-Kharj, Saudi Arabia; 4 Department of Medical Laboratories, College of Applied Medical Sciences, Shaqra University, Shaqra, Saudi Arabia

**Keywords:** chikungunya virus, emerging disease, neglected tropical disease, outbreak, vaccine development, vector

## Abstract

Chikungunya virus (CHIKV) is an arthropod-borne viral disease which spreads to humans by mosquito bites, inducing musculoskeletal pain and fever. CHIKV was endemic to the Indian Ocean region till 2004 that affected millions. It has been expanding its existence to non-endemic regions in Europe, the Middle East and the Pacific regions since 2004. Current fast CHIKV transmission scenario highlights the necessity of innovating control methods and devising novel diagnostic techniques. Conventional vector control measures lack efficacy as CHIKV vector evolves. Also, the existing assays used to detect CHIKV vary on sensitivity and specificity. This leads to mis-reporting or under-reporting of CHIKV cases especially in the endemic regions. This review discusses the CHIKV pathogenesis, an overview of various existing detection and the mitigation measures. Later, the challenges and limitations posed by these and how they can be subjugated by employing various simple and sustainable measures are emphasised. This review also suggests strategies to deploy novel systems in resource-limited settings to effectively address the infection and transmission of CHIKV disease.

## 1 Introduction

**FIGURE 1:**
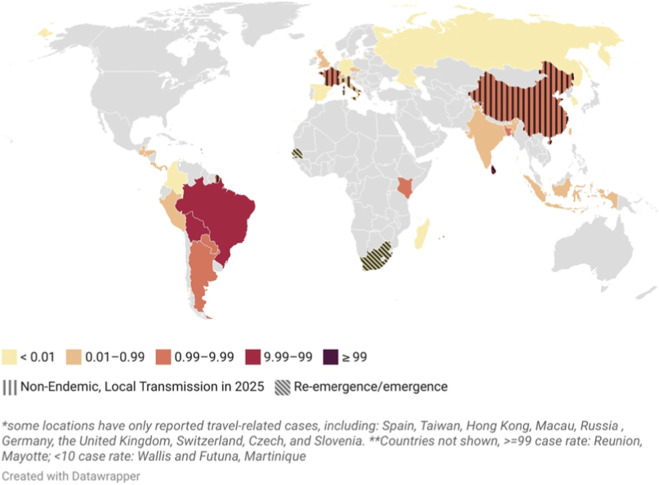
Geographical distribution and cumulative cases of CHIKV disease cases (Reproduced with permission from: https://bluedot.global/chikungunya-goes-global/; accessed on 20 November 2025).

Chikungunya, an arboviral disease spread to humans by *Aedes* mosquitoes, has emerged as a major public health concern. Since its initial reported outbreak in Tanzania in 1952, CHIKV has expanded worldwide and has been reported from over 110 countries (Vairo et al., 2019; Costa et al., 2023). The 2025 disease outbreak report of the WHO observed the global resurgence of CHIKV that affected 40 countries. 445,271 suspected and confirmed CHIKV cases and 155 related deaths were reported based on the data collected between 1 January and 30 September 2025 (WHO Disease outbreak report). It includes the major Chinese outbreak in Foshan City, Guangdong province on 9 July 2025, with over 10,000 confirmed cases by August 2025 (as per the CDC report). A surge in arboviral infection was witnessed with more than 38,000 suspected CHIKV infection cases in Cuba, a country that is struggling with strained healthcare system due to economic sanctions of the US (Taylor, 2025). CHIKV has been significant due to its rapid global spread, warranting a quick response (Figure 1).

The infected individual after the mosquito bite exhibits three infection stages: acute, post-acute and chronic (Nkoghe et al., 2012). Acute stage of about 21 days is characterised by symptoms like elevated fever, severe joint pain and swelling, headache, myalgia, rash and fatigue. High level of viraemia is typically observed between 5 and 10 days after infection, in this phase (Mourad et al., 2022). Acute stage is followed by the post-acute phase that may last up to 3 months, characterised by worsening of joint pain. The chronic phase follows the post-acute phase in certain infected individuals that may last for months to years. Characterised by chronic rheumatism, continued fatigue and severe joint pain, it hampers wellbeing and quality of life (Chang et al., 2018). As specific therapeutics were unavailable, acute CHIKV disease therapy is limited to supportive care and pain management with rest, oral hydration, analgesic and non-steroidal anti-inflammatory drug, while chronic cases are treated with non-steroidal anti-inflammatory drugs or anti-rheumatic drugs (Millsapps et al., 2022).

CHIKV of genus *Alphavirus* and family *Togaviridae* is a single-stranded, positive-sense RNA virus of one serotype and four lineages: East Central and South African, West African, Asian and Indian Ocean (Bartholomeeusen et al., 2023). A single vaccine could be effective to control Chikungunya as all the lineages are antigenically stable (Chua et al., 2016). Being vector-borne, CHIKV is transmitted primarily by two *Aedes* species, *A*. *aegypti* and *A*. *albopictus* (Pereira et al., 2022). Due to globalisation, urbanisation and altered environments, the CHIKV vector has expanded its geographic presence, raising the risk of sudden, volatile outbreaks significantly in the endemic and non-endemic countries (Giovanetti et al., 2023). Expanding CHIKV vector has elevated disease burden too, potentially leading to increased outbreaks, hospitalisation, vector control responses, and disrupted socioeconomic activities in affected regions (Giovanetti et al., 2023). Understanding CHIK global burden was critical in designing effective prevention and control strategy (Ribeiro dos Santos et al., 2025). Studies opine that CHIKV disease is upsetting due to its large-scale epidemic potential, characterised by severe and disabling joint pain that could lead to considerable productive man-hours and financial losses. In de Roo et al. (2024) estimated the global Chikungunya burden between 2011 and 2020, and concluded that CHIKV was responsible for 18.7 million cases from 110 countries with 1.95 million DALYs (disability adjusted life years). CHIKV caused about US$2.8 billion direct economic burden and about US$47.1 billion indirect burden over the past decade (de Roo et al., 2024).

A group of infectious diseases, neglected tropical diseases (NTDs) are caused by different aetiological agents (Mohapatra et al., 2025). The WHO put it under the NTD-list as the majority affected by CHIKV resided in resource-limited regions. Chikungunya was acknowledged as a major global health challenge in a WHO-published 2022 report (WHO NTD report). Being an NTD, its control and mitigation were essential to achieve sustainable development goal (SDG-3) that aims to end NTD outbreaks by 2030. The WHO consented for mitigation support like surveillance, sharing technical expertise and guidance in outbreaks reported regions. More focused and systematic studies to understand and reduce Chikungunya global burden, especially in marginalised countries was warranted to accomplish the goal.

Vaccine strategies research involving inactivated vaccine, live-attenuated vaccine, chimeric vaccine, recombinant vaccine, DNA vaccine, subunit vaccine and virus-like particle (VLP) are ongoing across the globe to develop safe and effective CHIKV vaccine (Edelman et al., 2000; Taylor et al., 2017; Muthumani et al., 2008; Metz et al., 2013; Roques et al., 2022). Valneva SE developed a live attenuated single-dose CHIK vaccine (Ixchiq-VLA1553) that was approved by the USFDA (United States Food and Drug Administration) in November 2023, after successful clinical trials. It became the world’s first licensed vaccine against a mosquito-borne disease (Weber et al., 2024) and, while various other potential candidates are on trials (Table 1), it currently is the only approved human vaccine in the US, EU and Canada. This vaccine must be deployed in CHIKV-endemic regions as a preventive measure with primary focus to control vector population (De Lima Cavalcanti et al., 2022). Chikungunya is ignored by the policymakers and public health agencies although it demonstrates higher morbidity and higher transmission as compared to other arboviral diseases like Dengue and Zika (Mohapatra et al., 2024).

**TABLE 1:** A list of different types of CHIKV vaccines currently at various stages of clinical trials.

Vaccine type	NCT number	Developer/sponsor	Vaccine	Clinical trial phase/status	Route of administration	Result
MV vectored recombinant CHIKV vaccine	NCT03028441	National Institute of Allergy and Infectious Diseases (NIAID)	VRC-CHKVLP059-00-VP	1/Completed	Intramuscularly	Data not publicly available^#^
	NCT02861586	Themis Bioscience GmbH	MV-CHIK-202	2/Completed	Intramuscular	Safe and effective in humans with no side effects, vaccinated individuals show 100% seroconversion, acceptable tolerability
	NCT03807843	Themis Bioscience GmbH	V184-006	2/Completed	Intramuscular	Data not publicly available^#^
	NCT03101111	Themis Bioscience GmbH	MV-CHIK	2/Completed	Intramuscular	Data not publicly available^#^
CHIKV-VLP based vaccine	NCT03992872	Bavarian Nordic	PXVX0317	2/Completed	Intramuscular	Immunogenic and well tolerated; no adverse effects reported
	NCT06007183	Bavarian Nordic	PXVX0317	3/Active	Intramuscular	Data not publicly available^#^
	NCT03483961	Bavarian Nordic	PXVX0317	2/Completed	Intramuscular	Safe, no side effects, log- lasting neutralizing abs till 2 years after vaccination
	NCT02562482	National Institute of Allergy and Infectious Diseases (NIAID)	VRC-CHKVLP059-00-VP	2/Complete	Intramuscular	Safe, immunogenic and well tolerated, no serious adverse effects, neutralizing abs in all dose groups
	NCT01489358	National Institute of Allergy and Infectious Diseases (NIAID)	VRC-CHKVLP059-00-VP	1/Completed	Intramuscular	Safe, immunogenic and well tolerated, no serious adverse effects, neutralising abs in all dose groups
	NCT07003984	Bavarian Nordic	Vimkunya	3/Recruiting	Intramuscular	Data not publicly available^#^
	NCT05072080	Bavarian Nordic	PXVX0317	1/Completed	Intramuscular	Safe, few individuals report self-limited adverse effects, elicits a rapid and robust immune response
	NCT05349617	Bavarian Nordic	PXVX0317	3/Completed	Intramuscular	Safe and well tolerated in 65years and older adults, high rate of protection within 2 weeks of PV
	NCT05065983	Bavarian Nordic	PXVX0317	2/Completed	Intramuscular	Data not publicly available^#^
CHIKV whole virus Inactivated vaccine	NCT04603131	Bharat Biotech International Limited	BBV87	1/Completed	Intramuscular	Data not publicly available^#^
	NCT06669208	Najit Technologies, Inc.	HydroVax-005	1/Recruiting	Intramuscular	Data not publicly available^#^
	NCT04566484	International Vaccine Institute	BBV87	2/3 Terminated	Intradermal	Data not publicly available^#^
Live-attenuated CHIKV vaccine	NCT03635086	Themis Bioscience GmbH	MV-CHIK	2/Complete	Intramuscular	Good immunogenicity, safe and acceptable tolerability
	NCT06106581	Valneva Austria GmbH	VLA1553	2/Completed	Intramuscular	Data not publicly available^#^
	NCT06973772	Butantan Institute	VLA1555 and Dengue 1,2,3,4 vaccine	3/Active	Intramuscular	Data not publicly available^#^
	NCT06028841	Valneva Austria GmbH	VLA1553	3/Withdrawn	Intramuscular in moderately immunocompromised adults	Data not publicly available^#^
	NCT03382964	Valneva Austria GmbH	VLA1553	1/Completed	Intramuscular	Data not publicly available^#^
	NCT04650399	Butantan Institute	VLA1553	3/Completed	Intramuscular	Safe and well tolerated, elicits seroprotective titers in all vaccinated groups, Induced neutralizing abs in 98% after 28 days PV
	NCT07133178	Valneva Austria GmbH	VLA1553	3/Active	Intramuscular	Data not publicly available^#^
	NCT04786444	Valneva Austria GmbH	VLA1553	3/Completed	Intramuscular	Data not publicly available^#^
	NCT04546724	Valneva Austria GmbH	VLA1553	3/Completed	Intramuscular	Safe and strong immune response, elicits virus neutralizing abs by 28 days PV
	NCT04440774	University of Oxford	ChAdOx1 Chik and ChAdOx1 Zika vaccines	1/Complete	Intramuscular	Data not publicly available^#^
	NCT06928753	Centre Hospitalier Universitaire de la Réunion	IXCHIQ	4/Recruiting	Intramuscular	Data not publicly available^#^
Simian ADV vectored vaccine.	NCT03590392	University of Oxford	ChAdOx1 Chik	1/Complete	Intramuscular	Data not publicly available^#^
CHIKV mRNA vaccine	NCT03325075	Moderna TX, Inc	VAL-181388	1/Completed	Intramuscular	Safe, well tolerated, elicited substantial and durable neutralising abs

^#^ as of 10.11.2025; MV: measles virus vectored; ADV: adenovirus; VLP: virus like particle; mRNA: messenger-RNA; PV: post vaccination; abs: Antibodies.

For every infectious disease, prompt and early laboratory diagnosis remains critical to reduce the associated morbidity, and this is true for CHIKV disease too. Various serological and molecular assays to detect CHIKV antigen, antibodies or viral RNA are currently in practice (Johnson et al., 2016). These include viral antigen detection by ELISA, IFA, rapid diagnostic test (RDT) and immune-blot methods. While plate reduction neutralisation test (PRNT) is used to quantify neutralising viral antibodies (Simo et al., 2023), molecular techniques like RT-PCR, rRT-PCR and isothermal amplification can detect viral RNA (Cardona-Trujillo et al., 2022). Clinical manifestations of Chikungunya are often identical to other mosquito-borne diseases prevalent in the regions, making diagnosis challenging. Thus, a clinical presentation alone is insufficient, and a differential diagnosis of patients who visited CHIKV endemic region was necessary. Despite being discovered more than 50 years ago, CHIKV is still posing global challenges with regard to disease prevention, monitoring, detection and management. This review provides an extensive summary of CHIKV detection and control challenges, the prevailing measures to contain this re-emerging human pathogen, and also suggests strategies betterment.

## 2 Methods

The veracity and replicability of this article was ensured through systematic search of several major academic databases, specifically focusing on PubMed, Scopus and the Web of Science. The search was limited only to the literature in English published between 1 January 2015 and 30 November 2025, ensuring the relevance of the reported findings. Key search terms coined from the core study objective included (but not limited to) “Chikungunya disease” OR “Chikungunya infection”; “Diagnosis of Chikungunya” and “Challenges”; “Diagnostic limitation of Chikungunya”; “challenges with Chikungunya”; and “Controlling Chikungunya”, with inclusion and exclusion criteria as described below.

Inclusion criteria: Peer reviewed, full-text articles published in English language that directly addressed the study objectives were included. Exclusion criteria: Preprints, conference abstracts and non-English vernacular language publications were filtered. A total of 92 literature were selected out of the 308 shortlisted searched articles. The full-text of the potentially relevant literature was reviewed, analysed, data extracted and qualitatively synthesised after an initial screening of the titles and abstracts, to identify comprehensive themes, trends and key findings. The process adhered to the standard reporting guidelines for narrative reviews (PRISMA) to minimise the bias and ensure transparency and replicability; however, less formal than the PRISMA guideline for systematic reviews (Page et al., 2021).

## 3 Pathogenesis of CHIK disease

Given the limited data regarding the nascent stages of CHIKV infection in human hosts, the succession of events comparable to that of the established ones for other arboviral diseases was considered. CHIKV reaches either the bloodstream or the skin tissue as an infected mosquito bites (Agarwal et al., 2016). Inside the dermis of the skin, CHIKV replicates in the fibroblast cells, but could also be supported by other cells like the dendritic cell, macrophage, endothelial cell, epithelial cell and keratinocytes (Labadie et al., 2010). CHIKV demonstrates a systemic dissemination to secondary sites of replication as it reaches the bloodstream (Ozden et al., 2007). CHIKV incubates for ∼1–12 days, after which the infected subject shows acute infection characterised by symptoms like fever, joint pain, headache, muscle aches, nausea and rash (Nkoghe et al., 2012). It was observed that the severity and duration may differ, and it can progress to crippling chronic symptoms in some (about 40%–80%) cases, like arthralgia and other complications that severely compromise the wellbeing (Chang et al., 2018).

CHIKV pathogenesis is a complex cascade of events involving both viral and host immune factors. Although the host immune response is critical in clearing the infection, it also contributes to CHIKV pathogenesis. As CHIKV is an RNA virus, the host’s immune system recognises it as a foreign antigen via specialised pattern recognition receptors (PRRs), such as toll-like receptors (TLRs). Studies showed that the absence of TLR3 led to the rise in disease severity in CHIKV infection in fibroblasts, and an SNP in TLR3 showed positive correlation with the disease severity (Her et al., 2015). Further, TLR4 is vital for CHIKV to attach and to enter host macrophages. Its inhibition leads to a lower viral titre, levels of viral E2 protein, and inflammatory bio-markers (Mahish et al., 2023). Type-1 IFNs is crucial in host defence against viral infection, and its deficiency enhances disease severity. PRRs and IFN signalling activation led to the induction of soluble factors like cytokines, chemokines and complement, which aggravate CHIKV immune responses and disease progression (Couderc et al., 2008).

The immune system responds with an intense inflammatory reaction when an individual is CHIKV-infected, flooding the body with high pro-inflammatory factors levels, like interleukins (IL-5, IL-6 and IL-18) and interferons (IFN-α and IFN-γ) (Wauquier et al., 2011; Ng et al., 2009; Chirathaworn et al., 2010), granulocyte colony-stimulating factor (G-CSF), granulocyte-macrophage colony-stimulating factor (GM-CSF), TNF-α (Chirathaworn et al., 2010; Rulli et al., 2011), and chemokines (CCL2, CCL3, and CXCL10) (Sokol and Luster, 2015; Chou et al., 2010). These molecules are essential as they act as signals to recruit immune cells at the infection site, initiate inflammation and organise long-term adaptive immune response against the virus (Chirathaworn et al., 2020; Wauquier et al., 2011; Nunes et al., 2023). The symptom severity and immunological biomarkers vary among the patients, and severe disease is correlated with high MCP-1/CCL2 and IL-6 levels and low IL-8 levels compared to mild cases (Lohachanakul et al., 2012). A study focused on the very early stages of infection found increased vital immunological indicators levels like MIG, CXCL10/IP-10 and complement C5a anaphylatoxin (Tanabe et al., 2019). A robust, early cytokine response, particularly pro-inflammatory cytokines like IL-6, during acute phase of the illness appears crucial for effective viral clearance and protection against chronic arthritis (Chang et al., 2018). Studies on chronic CHIKV phase observed persisting joint pain in patients, correlating with elevated biomarkers levels like IL-1β, IL-6, IL-8, MCP-1/CCL2, MMP-1 and MMP-3 (Ninla-Aesong et al., 2019), and GM-CSF (Chow et al., 2011). Animal studies showed that CD4+T lymphocytes played a pivotal role in CHIKV-induced inflammation, while the role of CD8^+^ T cells remained unclear. The precise mechanisms of tissue damage are not fully clear, but it is well-established that CHIKV infection triggers a potent inflammatory response. This process leads to high levels of pro-inflammatory cytokines like IL-1β and TNF-α, allegedly driving the characteristic tissue destruction especially in joints (Chirathaworn et al., 2020; Moreira et al., 2023; Tanabe et al., 2018).

CHIKV acquired mechanisms over the years to antagonise host antiviral immune response and evade the immune response. CHIKV nsP1 viral factor has the ability to regulate IFN-1 response, while nsP2 interferes with nuclear factor-kappa B (NF-κB) initiation pathways via MDA5/RIG-I to control pro-inflammatory cytokines (Webb et al., 2020; Bae et al., 2020). Further, CHIKV nsP2 could cause host cell shutdown, inhibiting host gene expression (Fros et al., 2015; Fros and Pijlman, 2016). Blocking the production of antiviral factors, ability of the virus to evade host immune response, is crucial in infection control. Deciphering the complex interplay between viral factors and host determinants, including host genetics and immune responses, is essential to understand CHIKV pathogenesis fully.

## 4 CHIKV diagnostic assays

**FIGURE 2:**
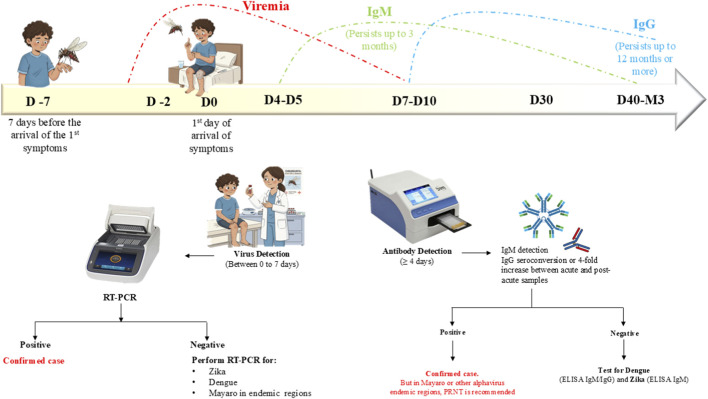
Time course of CHIKV viremia and immune response along with testing algorithm to detect the infection (Schwartz and Albert, 2010; National Center for Emerging and Zoonotic Infectious Diseases (U.S.). Division of Vector-Borne Diseases, 2016).

Based on the kinetics of viral infection markers like anti-CHIKV IgM/IgG antibodies, viral genome and virus-specific antigens, numerous laboratory-based diagnostic techniques are available. Diagnostic techniques to detect CHIKV in clinical samples include cell culture, serological methods like enzyme linked immunosorbent assay (ELISA), immunoblotting techniques, immunofluorescence assays (IFA), rapid diagnostic tests (RDTs and molecular methods like isothermal amplification (LAMP), conventional or standard RT-PCR, qualitative and quantitative real time RT-PCR assays (Johnson et al., 2016; Simo et al., 2023; Natrajan et al., 2019). Identifying viral RNA by rRT-PCR during acute phase (≤7 days of symptoms onset) and detecting CHIKV anti-IgG and IgM thereafter (>7 days) are recommended due to the kinetics of CHIKV viraemia and subsequent host immune response. rRT-PCR alone is primarily recommended from 0 to 5 days after the onset of symptoms. rRT-PCR and anti-CHIKV IgM are recommended between day 5 and 7, and CHIKV anti-IgG after 7 days of symptoms onset. A positive real-time RT-PCR (rRT-PCR) test result indicates active Chikungunya viral (CHIKV) infection whereas anti-CHIKV IgM antibodies detection suggests probable infection (Figure 2). Nucleic acid testing (NAT), conducted as a single-plex assay targeting CHIKV RNA or as a multiplex assay simultaneously screening for CHIKV, dengue virus (DENV) and Zika virus (ZIKV) RNAs, is highly specific and sensitive (Cardona-Ospina et al., 2022). However, a universally accepted molecular gold standard for CHIKV diagnosis is yet to be established. Accurate diagnosis of CHIKV during the time of an epidemic remains a formidable challenge.

## 5 Technical challenges in CHIKV detection

Detecting Chikungunya infection poses several challenges, primarily related to the clinical presentation, sample collection time, and limitations of the diagnostic assay adopted. The following subsections highlight these challenges.

### 5.1 Clinical superimpose with other arboviruses

The classical symptoms observed in Chikungunya cases include rapid onset of fever, severe debilitating arthralgia and rash that is commonly maculopapular (Zamarina et al., 2021).

Additionally, frequently reported symptoms include headaches and gastrointestinal issues such as nausea, vomiting, discomfort in the abdomen and anorexia. It has been noted that distinguishing CHIKV infections from other aetiologies like flaviviruses (DENV and ZIKV), alphaviruses, and non-viral agents like *Plasmodium*, *Rickettsia*, *Leptospira* and *Salmonella* requires more than just a clinical diagnosis (Acevedo et al., 2017). CHIKV cooccurs with many other arboviruses like DENV and ZIKA which also spread by *Aedes* mosquito. Further, Chikungunya symptoms are frequently indistinguishable from those in the acute phase of dengue. Joint pain and inflammation symptoms are similar to those of rheumatoid arthritis, which can lead to misdiagnoses and make the differentiation between the two disorders challenging (Chang et al., 2018).

This leads to delayed therapy, resulting in neurological (Webb et al., 2022) and cardiovascular (Webb et al., 2022) manifestations, thereby increasing the morbidity and mortality in the affected (Rama et al., 2024). Further, chronic CHIKV disease presents constant joint pain and arthritis-like syndromes, often confused with other chronic diseases. Clinical presentation of Chikungunya is complicated in the elderly patients above 65 years, with greater neurological complaints (Godaert et al., 2018). Establishing a rapid and accurate diagnosis to treat and manage Chikungunya, especially in children, co-morbid patients and the elderly, is paramount as it might lead to long-term complications in these individuals.

### 5.2 Limitation of time-dependent sampling

As discussed, diverse laboratory diagnosis techniques are in practice based on the kinetics of viral markers like anti-Chik antibodies, viral RNA and virus-specific antigens. CHIKV can be isolated from clinical samples during early infection using various insect and mammalian cell lines and by inoculating suckling mice (Natrajan et al., 2019). However, routine diagnosis does not require virus isolation as the process is expensive, time-consuming, technically demanding (requires BSL-3), and less precise than molecular diagnostic assays (Cardona-Trujillo et al., 2022). RT-PCR and real-time RT-PCR-based assays are more commonly employed these days to detect the CHIKV genome in clinical samples, being rapid and offering higher sensitivity and specificity. Molecular diagnostic assays like isothermal amplification (LAMP) are inexpensive but are often restricted to detecting CHIKV only during acute Chikungunya phase, i.e., in the first few days. The sensitivity of CHIKV RNA detection in serum samples collected from suspected patients declines between 4 and 7 days of onset, and thus detection of anti-CHIKV IgM in such samples is recommended. Serological assays like ELISA could detect CHIKV-specific IgM and IgG antibodies, but it cross-reacts with other alphaviruses and yield false positive/negative result. A significant portion of Dengue-confirmed patients often test positive for Chikungunya IgM antibodies, indicating considerable cross-reactivity or dual infection (Peeling et al., 2010). It necessitates accurate differential diagnosis that can be done using molecular techniques. Lack of precise point-of-care diagnostics for CHIKV delay the process and facilitates the transmission of CHIKV in the community. Delayed diagnosis of Chikungunya affects surveillance programmes and public healthcare efforts.

### 5.3 Challenges in resource-limited settings

Modern diagnostic assays like rRT-PCR provide accurate results but are expensive and need high skillset to perform the tests and analyse. The assay also needs sophisticated equipment and imported reagents which are challenging for the resource-constrained low- and middle-income countries and remote inaccessible regions. CHIKV RNA, being highly unstable, often needs maintaining proper cold chain. Such resources are typically scarce or non-existent mostly in low-resource lab settings, leading to CHIKV under-reporting. Degraded viral RNA during the transport impacts diagnosis and delays the implementation of control measures.

### 5.4 Analytical sensitivity and specificity

The sensitivity and specificity of an analysis refer to the ability of a test to detect or exclude target analyte under ideal lab conditions, while clinical sensitivity and specificity describe how well a test performs in real-world scenario. Studies have documented DENV and ZIKV co-circulation in endemic regions, particularly in the tropics and subtropics of the Americas and Asia. In practice, clinical performance can be influenced by factors such as co-circulation of related viruses like DENV and ZIKV, which may increase the likelihood of cross-reactivity and complicate the interpretation of the test result. Literature highlight that the antigenic similarities of DENV and ZIKV (closely related flaviviruses) can result in cross-reactivity in serological assays, making it difficult to ascertain the infection (Musso and Gubler, 2016; Wen and Shresta, 2019; Gaspar-Castillo et al., 2023). While a test might demonstrate high analytical accuracy, its clinical reliability is affected in co-infection scenario. Co-circulating viruses impact diagnosis accuracy and increase the risk of misinterpreted test results.

## 6 Operational challenges

The reemerged CHIKV is spreading to other adjacent areas owing to friendly environmental conditions for vectors, leading to higher infection cases. Global spread of CHIKV highlights the pressing need to develop and implement containment and control strategies to curb it, particularly in the face of scarce diagnostic equipments or technical inadequacy in resource-limited nations. Thus, it is imperative to improve the currently available diagnostic assays to accurately detect early and low CHIKV antigen levels, enabling efficient control and reduction of CHIKV infection. Additionally, these tests should enable accurate distinction between CHIKV infections and Dengue, Zika and other clinically significant arboviruses (Ribeiro et al., 2021). Multiplex tests that target several vector-borne diseases through PCR amplification would ensure greater specificity and enhanced sensitivity (Sani et al., 2025). There is a need to enhance point-of-care molecular testing to facilitate rapid, more affordable diagnosis (Hassan et al., 2025). Existing biomonitoring systems must be integrated with real-time data to detect CHIKV early, and thus manage its spread. Additionally, capacity building should include training the healthcare professionals and technicians in differential diagnosis of CHIKV, thereby enhancing surveillance initiatives and public health control strategies. Fast, low-cost, accessible and simple assay diagnostic technique that can precisely monitor local and global CHIKV infection progression may be developed, enabling informed decision making.

## 7 Challenges in CHIKV transmission and disease management

There are no therapeutics currently, and only one vaccine (IXCHIQ) is available worldwide to control CHIKV infection (Chen et al., 2024). For this, transmission control strategies for CHIKV primarily focus on vector control and self-protective measures to prevent mosquito bites, although each approach has limitations. Moreover, there are challenges in vector control measures, such as the lack of community engagement, resistance to insecticides, possible changes in vector behaviour, and a lack of appropriate experimental models for drug discovery or even vaccine efficacy testing, which are described below.

### 7.1 Lack of CHIKV-specific antivirals and/or vaccines

Chikungunya-infected subjects are managed through supportive care due to the unavailability of antivirals, which focuses primarily on relieving from symptoms like fever and myalgia by administering analgesics and physiotherapy to manage persistent joint pain; joint pain may continue for several weeks to months (Lozano-Parra et al., 2024; Watson et al., 2020). Research at the global scale to control CHIKV infection has prioritised drug repurposing and antivirals. Although numerous are at the trial stages, no specific candidate for public use is approved till date (Hucke and Bugert, 2020). VLA1553 (IXCHIQ), a live-attenuated preparation, has received regulatory approval only recently, as the first chikungunya vaccine (Ly, 2024; Weber et al., 2024). Many other vaccines with promising results in human trials include viral-vectored candidates (e.g., ChAdOx1), viral mRNA vaccines, inactivated-virus vaccines and virus-like particle constructs (Table 1). However, a few concerns even after promising trial results that need to be addressed are: 1) the efficacy of the developed vaccine in real-world settings; 2) the efficacy of the vaccine in the elderly and the immunocompromised; 3) the duration of the protection; 4) optimal manufacturing, stockpiling and supply of the vaccines in the endemic regions (Maure et al., 2024). The need to ensure equitable access and financing, especially in endemic and developing countries, is also felt (Ribeiro et al., 2025).

### 7.2 Vector adaptability

*Aedes* mosquito, the transmitting vector is well adapted to domestic breeding and lay eggs in water poodles in like roof gutters, unused water pots, standing water in flower pots, and even in discarded containers. These breeding sites allow them to thrive in high numbers for rapid disease transmission (Dharmamuthuraja et al., 2023; Wilke et al., 2020). Farmland, city house and open green space in suburban areas also provide breeding grounds for both *A*. *albopictus* and *A*. *aegypti*. These mosquito breeding hotspots are often overlooked due to their small size (Zhang et al., 2025; Liu et al., 2023). It is difficult to destroy the mosquito vector from its breeding ground by following conventional measures (Nie and Feng, 2023). Since these breeding hotspots are often found indoors or on private lands and are spread across in large numbers, the ‘search-and-drain’ drives are not enough. Clearing them requires regular efforts from both local authorities and households, and rapid re-infestation occurs if either side misses it (Dharmamuthuraja et al., 2023; Wilke et al., 2020). As *Aedes* mosquito can survive in urban or semiurban areas, regular community involvement in control measures could be encouraged (Perez-Guerra et al., 2023). The most effective way could be targeting the local breeding hotspots and regular surveillance or monitoring to reduce the rising mosquito numbers and minimising the overall transmission risk.

### 7.3 Challenges in community participation

Community involvement and awareness programmes to educate people about the household activities that promoted breeding of *Aedes* mosquitoes are crucial (Hossain et al., 2024). People may be encouraged to practice these measures regularly, but this plan alone may yield a short-term success only (Gopalan et al., 2021). These practices are curtailed due to the apprehension of additional financial burden over time (Perez-Guerra et al., 2023). Studies show that even the aware population continue the habit of storing water in open pot and not clearing the stagnant water from space like roof gutters. Further, the lack of trust among public health workers, their disease control experience and community involvement in control programmes are crucial in the control efforts. Studies conclude that community participation decreased when the messages did not fit with the local beliefs on disease transmission mechanisms (Hossain et al., 2024). Novel biological measures to control the vector can work only if the community accepts, understands the process, and involves (Sanchez-González et al., 2021). It is important to address these social obstacles and involve the community in strategising to achieve long-term success in vector control (Perez-Guerra et al., 2023). The need to develop viable economical strategies to control many vector-borne diseases like dengue and malaria besides chikungunya is felt, ensuring local ownership and sustainability (Sharma et al., 1995).

### 7.4 Global expansion of vector

The geographic spread of *Aedes* has expanded owing to factors like climate change, increased global travel and international trade. The mosquito is gaining access to regions that were previously unfavourable for their survival. This leads to the introduction of CHIKV in areas that were free of it. Climatic variations like increasing temperature and altered rainfall pattern have expanded the conducive mosquito breeding areas, thus increasing the transmission risk (Radici et al., 2025; Liu et al., 2025). *Aedes* mosquito has spread to new locations due to the rising commercial shipping and global trade of goods like used tires and plants. Continued introduction of goods and individuals and altered environmental conditions facilitate the expansion (Hu et al., 2025). Nations also confront the urgent threat of Chikungunya spread locally when the virus is introduced by infected travellers. Swift response and early disease identification become more challenging due to surveillance system inadequacy (Radici et al., 2025). With expanding mosquito habitats, control measures must go beyond relying solely on conventional measures like insecticide spraying. Nations need to prepare labs to detect the infected individuals swiftly, screen the travellers, and enhance mosquito surveillance system. Exchanging information between countries and utilising climate-aware risk assessments will aid identify fresh introduction and reduce future outbreak. To limit infection transmission, effective urban planning to minimise standing water would reduce mosquito breeding sites as globalisation increases and the temperatures continue to rise (Wilke et al., 2020).

### 7.5 Insecticide resistance

*A*. *albopictus* and *A*. *aegypti* have been resistant to insecticides like carbamates, pyrethroids and organophosphates over years (Malijan et al., 2024). This resistance may have developed through genetic mutations (Reyes-Perdomo et al., 2025; Keumeni et al., 2025) or enzymatic detoxification (Estep et al., 2025). Mosquitoes can develop resistance to insecticides that may vary across places. Applying only one type of insecticide may not work everywhere and is not reliable as a long-term solution. Thus, it is important to regularly monitor insecticide resistance patterns at regional level to keep the control efforts effective. This would necessitate choosing appropriate and alternative insecticide combination to ensure that the resistance development is minimised (Mendis et al., 2024). Biological control using *Wolbachia* is another effective way to address the challenge (Sanchez-González et al., 2021).

## 8 Addressing the challenges in CHIK control

A stronger integrated surveillance system that uses sero-surveys to trace community infection and employs multiplex molecular testing to detect multiple viruses simultaneously need to be established to effectively manage CHIKV infection control challenges. An integrated vector management strategy must be paired that modernises traditional methods with cutting-edge biological and genetic engineering, while incorporating environmental management and sustainable urban planning to eliminate mosquito breeding sites. Crucially, we must empower the public by providing clear, practical information and fostering community mobilisation for effective vector control, and at the same time, ensure test reliability and speed by updating labs and boosting international collaboration; ultimately, these combined efforts to reduce *Aedes* mosquitoes offer benefit by preventing other viral diseases, creating powerful defence against concurrent outbreaks when matched with better strategies to manage the surge.

## 9 Future perspectives

The future management of CHIKV challenges centres on a transition towards proactive, integrated strategy encompassing advanced diagnostics, modern therapeutics, and innovative vector control. Diagnosis will shift to rapid, multiplex point-of-care (POC) tests that can quickly differentiate CHIKV from similar arboviruses such as Dengue and Zika, supported by molecular surveillance to track mutations. Control efforts will be revolutionised by widely deploying new-generation vaccines (including mRNA and VLP platforms) for both routine and outbreak use, alongside developing specific antiviral drugs and monoclonal antibodies to treat acute infection and chronic arthralgia. Vector control will modernise through integrated vector management (IVM), combining traditional methods with biological (like *Wolbachia*) and genetic engineering, guided by climate-driven models to enable highly targeted, proactive intervention against *Aedes* and strengthen global preparedness against concurrent outbreaks.

## 10 Limitation

Although we have attempted to provide an overview of Chikungunya virus infection, with emphasis on its diagnostic challenges that hamper its mitigation, this overview has many potential limitations. First, the article sourcing process was restricted to online databases like Scopus, Pubmed and WOS, but no other databased were searched. Therefore, additional relevant studies might have been missed. Second, we included only studies published in English within a specified time frame, potentially excluding relevant literature published in other languages or earlier periods. This language and temporal constraints may narrow the scope of evidence and introduce publication bias. Third, the overall validation scope of this review remains low, given the heterogeneous methodologies and varying quality of the included studies. Finally, we have excluded articles published in preprint databases due to the lack of peer review. These limitations highlight the need for more diverse, comprehensive, and methodologically consistent future research to validate and expand upon the insights presented in this review.

## 11 Conclusion

The global fight against CHIKV requires a multi-faceted approach to address critical gaps in research and public health. We must prioritise understanding the viral replication cycle, its complex seasonal co-occurrence with Dengue, and the clear relationship with climate change to predict and manage outbreaks effectively. Crucially, advancing nanotechnology is the key to developing rapid, accurate and portable point-of-care diagnostics for quick clinical detection. While the recent USFDA-approved vaccine is a major success, exploration of alternative platforms like mRNA vaccines is necessary for robust global prevention. CHIKV transmission control hinges on implementing smart surveillance methodologies powered by AI tools and adopting climate-adaptive vector control strategies to target the *Aedes* mosquito effectively in a changing environment. We do understand that AI driven tools and portable nano-diagnostic assay are expensive, thus we emphasise on phase-wise implementation, pilot programmes and international collaboration to ensure feasibility in low-income countries. Thus, we require a combined strategy (from basic science to advanced tools) to mitigate acute and chronic CHIKV burden worldwide.
